# Corrigendum to: A Case Report of Capsular Contracture Immediately Following COVID-19 Vaccination

**DOI:** 10.1093/asjof/ojab041

**Published:** 2021-12-11

**Authors:** Richard J Restifo

In the article “A Case Report of Capsular Contracture Immediately Following COVID-19 Vaccination” by Richard J. Restifo, MD, published in *Aesthetic Surgery Journal Open Forum*, Volume 3, Issue 3, September 2021, https://doi.org/10.1093/asjof/ojab021, the author has asked for the video and video still image to be removed. The author regrets any inconvenience.

